# Consistency Is Key: A Secondary Analysis of Wearable Motion Sensor Accuracy Measuring Knee Angles Across Activities of Daily Living Before and After Knee Arthroplasty

**DOI:** 10.3390/s25133942

**Published:** 2025-06-25

**Authors:** Robert C. Marchand, Kelly B. Taylor, Emily C. Kaczynski, Skye Richards, Jayson B. Hutchinson, Shayan Khodabakhsh, Ryan M. Chapman

**Affiliations:** 1 Ortho Rhode Island, Wakefield, RI 02879, USA; 2 Department Kinesiology, University of Rhode Island, Kingston, RI 02881, USA; 3 Department of Electrical, Computer, and Biomedical Engineering, University of Rhode Island, Kingston, RI 02881, USA

**Keywords:** knee replacement, wearable motion sensor, IMU, ROM, telehealth, orthopaedics, kinematics

## Abstract

**Highlights:**

**What are the main findings?**

- Wearable motion sensors accurately measure knee angles before/after total knee arthroplasty (TKA).
- Error computing knee angles was equivalent regardless of activity completed.

**What is the implication of the main finding?**

- Wearable motion sensors can be reliably deployed before/after TKA.
- Future studies can leverage this technology to quantify preoperative/postoperative function.

**Abstract:**

Background: Monitoring knee range of motion (ROM) after total knee arthroplasty (TKA) via clinically deployed wearable motion sensors is increasingly common. Prior work from our own lab showed promising results in one wearable motion sensor system; however, we did not investigate errors across different activities. Accordingly, herein we conducted secondary analyses of error using wearable inertial measurement units (IMUs) quantifying sagittal knee angles across activities in TKA patients. Methods: After Institutional Review Board (IRB) approval, TKA patients were recruited for participation in two visits (*n* = 20 enrolled, *n* = 5 lost to follow-up). Following a sensor tutorial (MotionSense, Stryker, Mahwah, NJ, USA), sensors and motion capture (MOCAP) markers were applied for data capture before surgery. One surgeon then performed TKA. An identical data capture was then completed postoperatively. MOCAP and wearable motion sensor knee angles were computed during a series of activities and compared. Two-way ANOVA evaluated the impact of time (pre- vs. post-TKA) and activity on average error. Another two-way ANOVA was completed, assessing if error at local maxima was different than at local minima and if either was different across activities. Results: Pre-TKA/post-TKA errors were not different. No differences were noted across activities. On average, the errors were under clinically acceptable thresholds (i.e., 4.9 ± 2.6° vs. ≤5°). Conclusions: With average error ≤ 5°, these specific sensors accurately quantify knee angles before/after surgical intervention. Future investigations should explore leveraging this type of technology to evaluate preoperative function decline and postoperative function recovery.

## 1. Introduction

Total knee arthroplasty (TKA) is one of the most effective and successful elective interventions currently available [1,2]. Following TKA for treatment of knee osteoarthritis (OA), patients frequently experience improved satisfaction and range of motion (ROM) during many activities [3,4,5,6,7,8,9]. Accordingly, knee ROM is often utilized as a metric of recovery following TKA. The current standard of care for evaluating knee ROM post-TKA is repeated clinical visits wherein sagittal knee ROM (i.e., flexion/extension) is evaluated via a goniometer in static or quasi-static poses. Although this approach affords ease of implementation, using goniometry to evaluate postop knee function is distinctly limited (e.g., low accuracy, discrete measurements) [10,11,12,13,14,15].

The advent of wearable motion sensors that contain inertial measurement units (IMUs) has facilitated the ability to continuously quantify knee ROM during a variety of activities before and after TKA. Our laboratory and others have effectively leveraged this approach to accurately assess joint kinematics, including knee ROM, both pre- and post-operatively, in TKA patients [16,17,18,19,20,21,22,23]. However, most studies evaluating knee ROM in TKA populations have utilized research-grade wearable motion sensors that are not frequently deployed in routine clinical practice. In the last five years, commercially available wearable motion sensors containing IMUs have increased prevalence for quantifying knee kinematics before/after clinical intervention. Preliminary evaluation of these systems, including work in our own laboratory, has indicated acceptable accuracy in quantifying sagittal knee kinematics when commercially available wearable sensor systems are appropriately deployed by the end-user [24,25,26]. However, these prior efforts either only evaluated healthy individuals [26], did not utilize gold-standard comparisons [24], or failed to compare across a range of activities [25]. Given knee ROM demands differ greatly across different activities (e.g., high versus low flexion), analyses are needed to evaluate clinically deployed IMU systems versus gold-standard optical motion capture (MOCAP), comparing error properties across a wide range of relevant activities of daily living (ADLs) [27,28,29,30].

Accordingly, we assessed the accuracy of one clinically deployed IMU-based wearable motion sensor system calculating sagittal knee angles versus optical MOCAP in TKA patients, comparing errors across a range of ADLs. Because this technology is deployed continuously (i.e., all day long), it needs to be accurate across a wide range of ADLs. Thus, our aim was to compare errors across activities common during rehabilitation following TKA and those frequently experienced during daily life. We hypothesized that (1) error would be less than current clinically accepted error thresholds (≤5°) across all activities [24,31,32], (2) error would be significantly greater during activities demanding higher knee ROM (e.g., sit-to-stand), and (3) error would be significantly greater during activities with increased knee ROM variability (e.g., timed up-and-go).

## 2. Materials and Methods

### 2.1. Enrollment and Participants

Given temporal and financial limitations, enrolling and capturing an entirely new sample of TKA patients, the data presented herein consist of secondary analyses on previously captured and published data [25]. However, a brief summary of the original methodological procedures is contained herein. During initial data collection, our Institutional Review Board (IRB) approved enrollment of n = 20 participants undergoing unilateral primary TKA, who were prospectively enrolled from a single surgeon’s clinic (*n* = 5 lost to follow-up). Exclusion criteria included ages outside of 50–80, Body Mass Index (BMI) > 40, prior TKA, musculoskeletal and/or neuromuscular dysfunction outside of knee OA, discomfort using smartphones and wearable sensors, and history of adhesive-related skin irritation.

**Figure 1:**
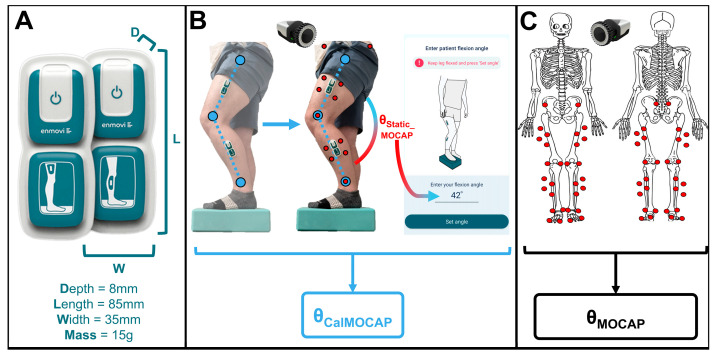
Measurement techniques including (**A**) wearable motion sensors and associated physical dimensions, (**B**) wearable motion sensor attachment and calibration using optical MOCAP angles, and (**C**) optical MOCAP markers.

Eligible participants consented at the biomechanics laboratory ~1-week pre-TKA. Participants then completed a sensor-use instruction session (Figure 1A; MotionSense, Stryker, Mahwah, NJ, USA) to ensure proper wearable motion sensor system use. Subsequently, sensors were applied to the impacted lower extremity per manufacturer instructions via supplied adhesive patches. IMU sensor calibration was then completed according to manufacturer recommendations (standing with the impacted leg elevated on a 10 cm block and inputting that pose’s sagittal knee angle [24,25]). In our previously published study [25], goniometric calibration angles were significantly different than MOCAP-measured calibration angles. Accordingly, herein we used MOCAP-measured sagittal knee angles during the calibration pose for calibrating the wearable sensor system (Figure 1B).

### 2.2. Study Flow (Figure 2)

Approximately 1 week before surgery, preoperative data were captured, followed by robot-arm assisted TKA (Mako Surgical Corporation, Weston, FL, USA) by a single surgeon via the medial parapatellar approach. The same make/model implant (Triathlon Cementless CR TKA, Stryker, Mahwah, NJ, USA) was used in all participants. Standard outpatient physical therapy at self-selected clinics was then completed by all participants. Approximately 6 weeks post-TKA, participants returned for an equivalent postoperative data capture with IMUs self-applied.

**Figure 2:**
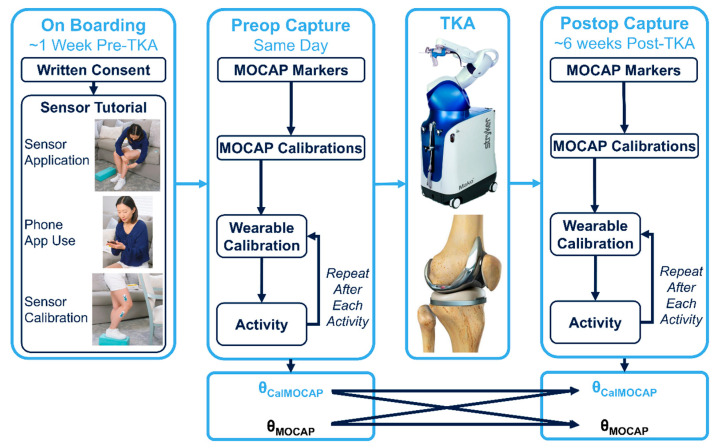
Study flow including pre-TKA onboarding, pre-TKA data capture yielding IMU (*θ_CalMOCAP_*) and MOCAP angles (*θ_MOCAP_*), surgery, and identical post-TKA data capture.

#### 2.2.1. Biomechanics Instrumentation

MOCAP calibration was completed per manufacturer recommendations (8 M3 Miqus Cameras, Qualisys AB, Goteborg, Sweden; 60 s, f_s_ = 100 Hz). A validated optical MOCAP marker set was utilized (Figure 1C) [33,34,35], and all 3D MOCAP marker position data were captured/stored in Qualisys Track Manager (QTM) at 100 Hz during calibration poses and activities [34]. Wearable sensors (6DOF, f_s_ = 50 Hz) were then applied to the impacted lateral thigh/shank per manufacturer’s instructions via supplied adhesive patches. Visual inspection of sensor placement ensured appropriate attachment. Wearable data (3DOF accelerometer and gyroscope) were then wirelessly streamed from both the thigh/shin sensor to a phone via Bluetooth during calibration poses and all activities. MOCAP marker positions in 3D and sagittal knee angles computed by the wearable sensors were temporally synchronized and stored for post-processing. MOCAP marker data were converted to .c3d for import into Visual3D (V3D, C-Motion, Inc., Gaithersburg, MD, USA) for MOCAP kinematic analyses to output sagittal angles of the impacted knee. Sagittal knee angles computed by the wearable sensors were converted to .xls for comparison.

MOCAP calibrations (static and dynamic) [36] created skeletal models with pelvis/lower extremity bony segment coordinate systems as described in previous publications [25,37,38]. Subsequently, wearable sensor calibration was conducted as in previous studies (Figure 1B) [24,25,26]. To ensure the most accurate calibration knee angles, MOCAP-computed sagittal knee angles (*θ_Static_MOCAP_*) were input into the associated cellphone application for wearable calibration. All subsequent wearable sensor algorithms were created by the manufacturer and are contained either directly on-board the wearable sensors or are contained in post-processing in their mobile application. During this calibration, both shin/thigh sensors leverage a Madgwick filter to compute their respective pitch/roll [24,25,39]. Assuming a pin-joint for the knee, roll/pitch for both sensors (*θ_Shin/Thigh_*, *Ψ_Shin/Thigh_*) were converted to rotation matrices (Equation (1)) followed by rotating the shin sensor data into thigh sensor coordinates (*R_Shin__→Thigh_*; Equation (2)). One angle (*α_IMU_*) was computed between the two rotation matrices (Equation (3); *R_Shin__→Thigh_* and *R_Thigh_*). To account for sensor-to-bony-segment misalignment, the MOCAP angle computed during calibration (*α_MOCAP_Calibration_*) was subtracted from the angle computed by the wearables during calibration (Equation (4); *θ_Offset_*). Then, during all activity trials, this offset angle was subtracted from the angles computed by the wearable sensors for all time (Equation (5)), and the final sagittal knee angles (α_Knee_) were exported for analyses.

(1) $$R_{Shin,Thigh}=\left[\begin{array}{ccc} \cos\theta_{Shin,Thigh} & \sin\Psi_{Shin,Thigh}\sin\theta_{Shin,Thigh} & \cos\Psi_{Shin,Thigh}\sin\theta_{Shin,Thigh}\\ 0 & \cos\Psi_{Shin,Thigh} & -\sin\Psi_{Shin,Thigh}\\ -\sin\theta_{Shin,Thigh} & \sin\Psi_{Shin,Thigh}\cos\theta_{Shin,Thigh} & \cos\Psi_{Shin,Thigh}\cos\theta_{Shin,Thigh} \end{array}\right]$$

(2) $$R_{Shin\to Thigh}=R_{Thigh}\cdot R_{Shin}^{T}$$

(3) $$\alpha_{IMU}=\cos^{-1}\left(\frac{trace\left(R_{Shin\to Thigh}\right)-1}{2}\right)$$

(4) $$\theta_{Offset}=\alpha_{IMU\_Calibration}-\alpha_{MOCAP\_Calibration}$$

(5) $$\alpha_{KNEE}=\alpha_{IMU\_Activity}-\theta_{Offset}$$

#### 2.2.2. Activities (Figure 3)

In addition to calibrations, participants completed three trials of sit-to-stand, standing knee bends, seated heel slides, seated march, long arc quads exercise, wall slides, timed-up-and-go test, and self-selected speed treadmill gait. After completing three trials of one activity, wearable sensor calibration was re-performed before the next activity.

**Figure 3:**
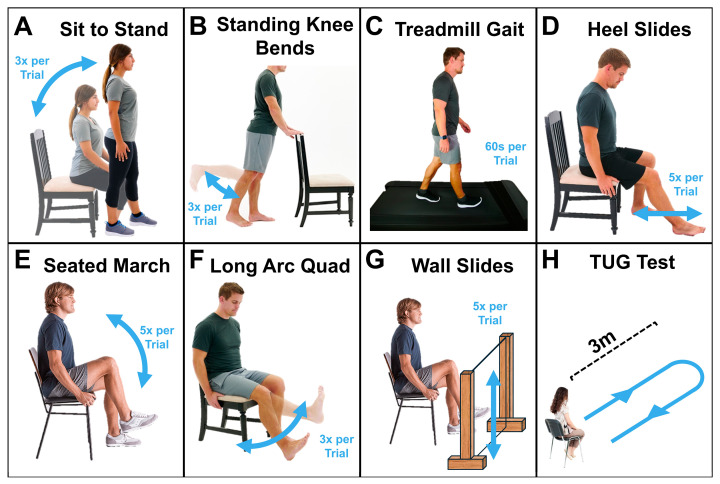
Activities completed by participants, including (**A**) sit to stand, (**B**) standing knee bends, (**C**) treadmill gait, (**D**) heel slides, (**E**) seated marches, (**F**) long arc quadriceps exercise, (**G**) wall slides, and (**H**) the timed-up-and-go test [25].

### 2.3. Data Analyses

#### 2.3.1. QTM and V3D Processing

MOCAP markers were manually labeled during static/dynamic calibration to create an automatic identification of marker (AIM) model to automatically label/track subsequent files. The AIM model was then applied to all activity files. Fully tracked/labeled static calibration and activity data were exported (.c3d) to V3D for further processing/final analyses. Static .c3d file was then used to create a base skeleton with a CODA pelvis [40,41], femora defined from hip joint centers to femoral epicondyle markers, and tibiae defined from femoral epicondyle markers to malleolar markers. Dynamic shin/thigh tracking during activities was accomplished via respective marker clusters throughout activities.

The static skeletal model was then applied to all activity files. MOCAP marker data were low-pass filtered (4th order Butterworth; f_cutoff_ = 6 Hz) to remove noise and interpolated between marker gaps (≤10 frames, 3rd order polynomial least-squares). Processed MOCAP marker data were converted to bony segments with 3D knee angles calculated via V3D algorithms, computing the relative orientation between the tibiae and femora via Cardan sequencing (X-Y-Z). Flexion and extension were defined as positive and negative values, respectively, and were exported for comparison to the wearable motion sensor calculated angles. Outcome variables were impacted knee sagittal angles (*θ_MOCAP_*) during all activities. *θ_MOCAP_* was then down-sampled to 50 Hz to match wearable motion sensor data capture frequency and converted to .xls format for statistical analyses.

#### 2.3.2. Wearable Sensor Processing

IMUs contained in the wearable motion sensor system measured 3DOF acceleration and gyroscopic data for both shin/thigh sensors. These data were then wirelessly streamed to a mobile phone where sagittal knee angles were computed as previously described (see Section 2.2.1). Utilizing calibration information, these angles automatically accounted for associated sensor-to-segment offset angles computed via MOCAP during wearable sensor calibration (*θ_Static_MOCAP_*). Final IMU-computed sagittal knee angles during all activities were exported, generating *θ_CalMOCAP_*, and placed in .xls files for statistical analyses (Figure 1B).

#### 2.3.3. Statistics

Statistics were then completed in SPSS (SPSS Statistics 29.0.1.0, IBM Corp., Armonk, NY, USA). Our previous publication contains all descriptive statistics for the enrolled cohort [25]. The difference between *θ_MOCAP_* and *θ_CalMOCAP_* was continuously calculated during all trials of all activities for all participants. This difference was averaged within each activity for each participant before/after TKA. Additionally, the difference between *θ_MOCAP_* and *θ_CalMOCAP_* was quantified at the local maxima (e.g., peak swing flexion during gait, etc.) and local minima (e.g., peak knee extension during heel slides, etc.) during all trials of all activities for all participants. A Shapiro–Wilk test (*α* = 0.05) was leveraged to assess data normality on average error as well as average error at local maxima/minima. Any metric failing the normality assessment had all statistical outliers (>3 standard deviations away from the mean) removed and a repeat Shapiro–Wilk assessment completed. Subsequently, the absolute value of all error metrics (average, local maxima, local minima) was calculated for each participant within each activity. Final measures for statistical assessments were average absolute value difference and average absolute value difference at the local maxima/minima for each activity and all participants pre-/post-TKA. On normally distributed average absolute value error data, a two-way ANOVA (*α* = 0.05) with Tukey post hoc tests was completed to assess the impact of time (pre-TKA, post-TKA) and activity on average absolute value error. Given that no significant difference was noted for data-capture timepoint (see Section 3.3), average absolute value error at local maxima/minima was combined across data-capture timepoints. Then, a two-way ANOVA (*α* = 0.05) with Tukey post hoc tests was completed to assess the impact of error metric (maxima vs. minima) and activity on average absolute value error at the local maxima/minima. Additionally, cross plots of MOCAP-computed sagittal knee angles versus IMU-computed sagittal knee angles were generated for each activity including all participant’s performance during all trials of that particular activity. Linear regressions were completed for each activity to generate coefficients of determination (R^2^) and linear regression equations.

## 3. Results

### 3.1. Participant Characteristics

All descriptive data for this cohort are contained in our prior publication [25]. In brief, this cohort was typical for patients undergoing TKA (average age = 69 years, average BMI = 30 kg/m^2^, balanced sex/surgical side). All participants completed pre-TKA data captures, with five participants lost to follow-up and two participants experiencing sensor failure in the middle of the post-TKA data capture. This resulted in n = 20 pre-TKA and n = 13 post-TKA data captures. Preoperative data captures occurred on average 5 days before TKA, and postoperative data captures occurred on average 33 days after TKA.

### 3.2. Example Data (Figure 4)

Example data for each task shows that wearable motion sensor-computed angles visually matched well with MOCAP-computed angles. Notable departures between wearable motion sensor- and MOCAP-calculated angles occurred at terminal ROM for particular tasks. For example, wearable motion sensors frequently overestimated knee flexion at maximal knee ROM during the sitting portions of sit-to-stand, swing phase of treadmill gait, and peak knee flexion during seated march activity (arrows). Additional periods of time where wearable motion sensors departed from MOCAP at terminal ROM were underestimations of MOCAP angles during the standing portion of sit-to-stand task and during full knee extension during the heel slide activity (stars).

**Figure 4:**
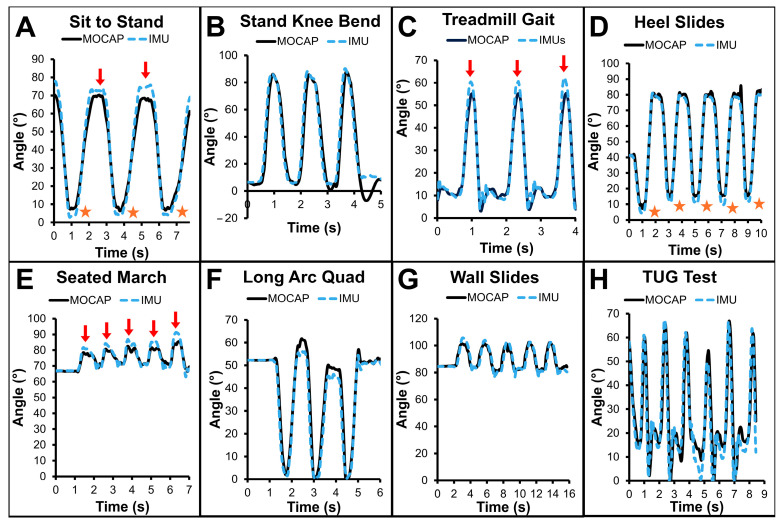
Exemplar sagittal knee angles computed by optical MOCAP (solid black line) and IMUs (dashed blue line) for all activities including (**A**) sit to stand, (**B**) standing knee bends, (**C**) treadmill gait, (**D**) heel slides, (**E**) seated marches, (**F**) long arc quadriceps exercise, (**G**) wall slides, and (**H**) the timed-up-and-go test. Differences between IMU and MOCAP angles were noted during specific activities at moments of peak flexion (arrows) and moments of peak extension (stars).

### 3.3. Results—Cohort Error Metrics

Shapiro–Wilk results are contained in Table 1. All three error metrics were initially non-normally distributed (*p* < 0.001 for all) with both average error and local maxima error initially moderately, negatively skewed (−1 < γ < −0.5) and local minima error slightly, positively skewed (γ < +0.5). Kurtosis further indicated that all error metrics were initially non-normal, leptokurtic distributions (κ > 0). Following removal of outliers (n = 12, n = 7, and n = 8 for average error, local maxima error, and local minima error, respectively), all three error metrics were normally distributed (*p* > 0.3 for all metrics) with minimal skew (|γ| < 0.16 for all metrics) and kurtosis (|κ| < 0.17 for all metrics).

**Figure 5:**
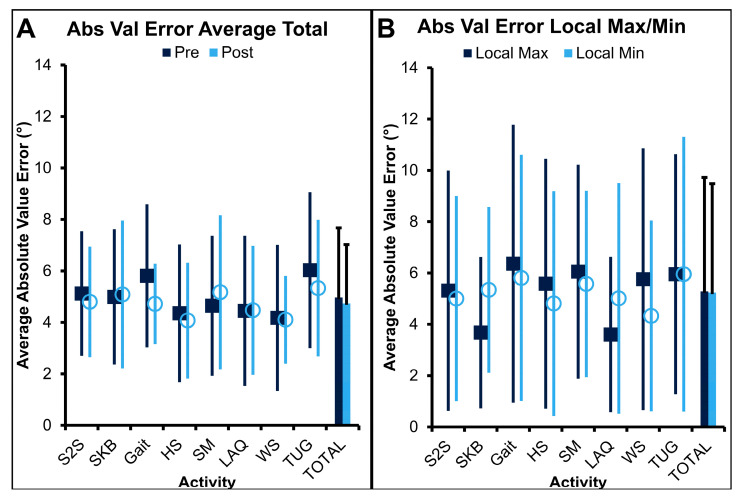
Average absolute value error ± standard deviation: (**A**) pre-TKA (dark blue squares) vs. post-TKA (light blue circles) across activities and (**B**) at local maxima (dark blue squares)/minima (light blue circles) by activity for all participants. No significant differences were noted across any activity, data capture timepoint, or error metric. S2S = sit-to-stand, SKB = standing knee bend, HS = heel slides, SM = seated march, LAQ = long arc quadriceps exercise, WS = wall slides, TUG = timed-up-and-go test.

Following normality assessments, the absolute value of all error metrics for all subjects/activities/trials was calculated. For average error, the two-way ANOVA (Figure 5A) showed no significant interaction between activity and data capture timepoint (*F* = 0.267, *p* = 0.966). Additionally, there was no main effect of data capture timepoint (*F* = 0.425, *p* = 0.515). This implies there was no difference between errors during pre-TKA data captures (dark bar; 5.0 ± 2.7°) compared to post-TKA data captures (light bar; 4.7 ± 2.3°). Critically, on average, error was below clinically acceptable thresholds (≤5°) during all data captures. Given no differences were noted pre-TKA vs. post-TKA, data were combined for the remaining analyses. There was also no significant impact of activity on error (*F* = 1.150, *p* = 0.333). This indicates that no activity had greater error than any other. Although not statistically significantly greater, treadmill gait (5.4 ± 2.4°) and TUG test activities (5.7 ± 2.8°) showed the greatest error on average and slightly exceeded the 5° clinical threshold. In contrast, all other activities were at or below 5° error including heel slides (4.2 ± 2.4°), long arc quadriceps exercise (4.5 ± 2.7°), sit-to-stand (5.0 ± 2.2°), standing knee bends (5.0 ± 2.6°), seated marches (4.8 ± 2.7°), and wall slides (4.1 ± 2.4°).

Two-way ANOVA on average absolute value error at local maxima/minima (Figure 5B) highlighted no significant interaction between activity and error metric (local maxima vs. local minima) (*F* = 0.924, *p* = 0.487) nor were there main effects of error metric (*F* = 0.023, *p* = 0.880) or activity (*F* = 1.1434, *p* = 0.189). As such, there was no differences between the local maxima average absolute value error (dark bar; 5.3 ± 4.4°) and local minima average absolute value error (light bar; 5.2 ± 4.2°). Errors at both local maxima/minima slightly exceeded the clinically acceptable error threshold of 5° on average. Interestingly, average absolute value error at local maxima/minima was at or below that threshold for long arc quad, standing knee bend, and wall slide activities (4.4 ± 3.9°, 4.5 ± 3.2°, and 5.0 ± 4.5°, respectively). The remainder of the activities exceeded 5° of error on average at local maxima/minima (sit-to-stand = 5.2 ± 4.3°, gait = 6.0 ± 5.1°, heel slide = 5.3 ± 4.6°, seated march = 5.8 ± 3.9°, timed-up-and-go test = 5.8 ± 4.9°).

**Figure 6:**
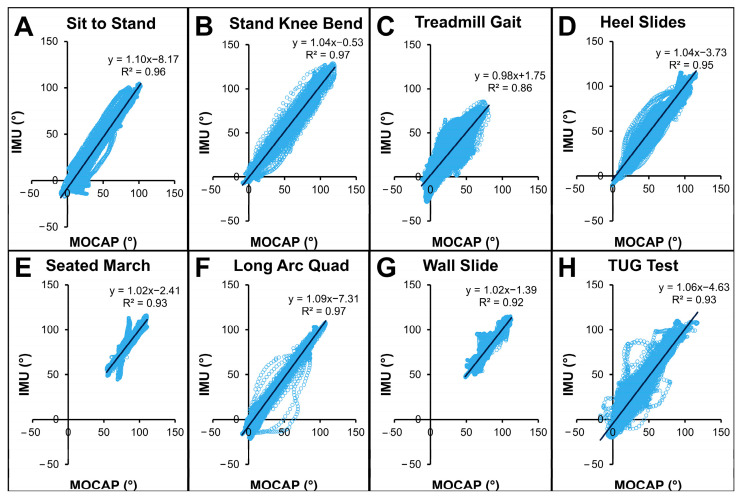
Cross plots for all activities ((**A**) sit to stand, (**B**) standing knee bends, (**C**) treadmill gait, (**D**) heel slides, (**E**) seated march, (**F**) long arc quadriceps exercise, (**G**) wall slides, and (**H**) timed up and go test) showed good correspondence between MOCAP-computed and IMU-computed sagittal knee angles.

If IMUs perfectly matched MOCAP, linear regressions would show R^2^=1.0 with linear fit equations containing slopes of 1.0 and zero intercept. Cross-plots (Figure 6, Table 2) and linear regressions showed coefficients of determination were all ≥0.86 regardless of activity, with treadmill gait the lowest (R^2^ = 0.86) and both standing knee bend and long arc quad activities the highest (R^2^ = 0.97). Linear regression equations all had slopes within 10% of ideal (range: 0.98–1.10). Standing knee bend, treadmill gait, heel slide, seated march, wall slide, and TUG test all had intercepts within clinically acceptable thresholds (≤5°). In contrast, sit-to-stand and long arc quad activities had intercepts beyond that threshold, with IMUs underestimating sagittal knee angles by 8.2° and 7.3°, respectively.

## 4. Discussion

### 4.1. Summary

Wearable motion sensors have afforded clinical-patient teams the ability to quantify a variety of metrics outside of well-controlled settings. In orthopaedics, ROM measurements have become increasingly important for quantifying function before and after interventions. For TKA, these measures were historically captured via high error (goniometry) or high cost (MOCAP, videoradiography) methods. Wearable motion sensors now offer a lower-cost option for calculating accurate ROM measures in free-living settings. Many of these sensors have remained prevalent in academic research settings but have not been commonplace in routine clinical care. Recently, however, several commercially available wearable sensors have made it to the marketplace and are being routinely deployed in the clinic. We previously validated one of these systems compared to the gold-standard optical MOCAP [25]. However, our initial analyses did not investigate differences across a variety of relevant activities. Given that these devices are intended to continuously quantify ROM across a wide range of activities in patient-selected settings, error assessment needs to evaluate IMU performance across activities. Accordingly, herein we conducted a secondary analysis of previously captured data to investigate error differences across activities in patients undergoing TKA.

### 4.2. Example Subject Data (Figure 4)

Time series data appeared well-matched between IMU-computed and MOCAP-computed sagittal knee angles across activities. This is similar to a number of previous studies [42,43]. In general, the shape and time-sequence of all data were appropriate; however, there were distinct periods of time where IMU angles departed from MOCAP angles. Of note, this tended to occur at peak ROM in either direction, with moments of divergence occurring at transition knee angle apices. Rhudy et al. found a similar result wherein knee flexion was predominantly well-matched but departed from optical MOCAP measures during peak flexion and extension [44]. This could happen for a number of reasons including soft tissue deformity at end ROM or soft-tissue noise induced by transitioning from moving toward flexion to moving toward extension (or vice versa).

### 4.3. Discussion—Cohort Error Metrics

Importantly for assessing the error characteristics of this sensor system, these analyses confirmed our first hypothesis that the average absolute value error would be under clinical standards (≤5°). Specifically, we found that the error was less than or equal to that threshold for both pre-TKA (5.0°) and post-TKA (4.7°) data captures. This indicates that this wearable sensor system is an effective and valuable tool for quantifying sagittal knee motion in TKA patients before and after surgery. This is similar to the results by several additional groups assessing a number of activities in healthy individuals as well as patients after TKA [45,46]. Moreover, this is a similar result to groups evaluating knee kinematics using a variety of different IMU sensor systems [19,47,48,49]. In the future, wearable motion sensors could be deployed to evaluate preoperative function (and associated disease severity) as well as assess postoperative function (and associated recovery trajectory after intervention). Interestingly, however, several activities exceeded this clinical threshold including treadmill gait and the TUG test. This may imply that quantifying sagittal knee motion using these sensors during those activities may not be advised. However, the error during both tasks was within 1° of the established threshold, which is likely not clinically relevant. Future investigations should query differences in function/outcomes in TKA patients to assess whether errors exceeding 5° are clinically relevant.

Secondarily, we hypothesized that error would be significantly greater during high ROM activities and during activities that require greater ROM variability. Interestingly, we found no significant impact of activity on error, indicating errors were not different across activities. As such, our second and third hypotheses must be rejected. Despite no statistical differences across activities, errors did increase during activities that traversed a wider ROM including treadmill gait and the TUG test. Although this could have resulted due to the larger ROM variability required during these tasks, the increased speed of these activities (relative to the other activities) is more likely the causative factor. Additionally, although not statistically significant, the regression analyses showed that sit-to-stand and long-arc quadriceps activities had intercepts that exceeded the 5° clinical acceptability threshold for error. Although we cannot definitively state why this is the case, one possible explanation for higher error herein could be soft tissue deformity during seated postures or increased frontal/transverse kinematics during these activities that add to the error stack-up. As such, caution is urged when leveraging these sensors during activities that are predominantly performed in a seated position. Despite these findings, given that no activity had significantly more error than others and error was on average under clinically acceptable thresholds, additional investigations can now explore the use of these sensor systems to evaluate post-TKA recovery in real time.

### 4.4. Limitations

Although this study pushes the use of clinically deployed wearable motion sensors forward, there are distinct limitations we should note. First, this was a secondary analysis of previously captured/published data. Additionally, we only quantified knee ROM and error using IMUs in a single cohort of individuals from one surgeon’s clinical practice who all received the same TKA implant. As such, extrapolation to other patients, surgeons, or implant manufacturers/models may not be possible from the present analysis.

In addition, we only evaluated a single IMU system. While other systems and sensor types do exist, this secondary analysis only permitted evaluation of one sensor system. As such, inferring this result in a different system may be ill-advised. Combined with this limitation, we assessed a limited subset of activities. While error results herein were promising, there are additional activities that TKA patients likely perform (e.g., stairs, squatting) that may have different error characteristics than those quantified in the present study. As such, future studies should endeavor to quantify the error of a broader range of tasks than presented herein. An additional limitation of the present study is evaluating errors in a well-controlled laboratory setting. Although the results herein are promising, previous work from our laboratory has shown that TKA patients move differently in free-living settings compared to well-controlled clinical/laboratory settings [20]. As such, subsequent studies should evaluate the error properties present in wearable motion sensor systems while patients perform activities outside of well-controlled lab/clinic environments. Finally, the present secondary analysis evaluated the error properties of this IMU system when sensors were applied according to manufacturer recommendations. Additional errors may be present in the final use case if patients fail to apply the sensors properly. And while error properties were acceptable when sensors were utilized appropriately, future studies should evaluate the impact of sensor to segment misalignment on final sagittal knee angle computations.

## 5. Conclusions

Herein, we conducted a secondary analysis of the error present using wearable motion sensors to quantify sagittal knee angles from patients before and after TKA. When following manufacturer-recommended procedures, the error was acceptable (≤5°) both before and after TKA. While the error was slightly greater in activities with higher velocity, the average error was equivalent across activities both before and after surgical intervention. Accordingly, this sensor system is reliable for quantifying sagittal knee angles in patients undergoing TKA. Despite success, limitations of a relatively small sample size (n = 20 pre-TKA, n = 13 post-TKA participants), using a single surgeon, and only assessing a single clinically deployed wearable sensor system warrant caution in generalizing the findings herein to broader applications. Future investigations should evaluate the use of this type of wearable motion sensor for quantifying disease severity before TKA and recovery following TKA.

## Figures and Tables

**Table 1:** Results of Shapiro–Wilk normality assessments.

Error Metric	Test #1				Test #2		
	*p*	Skewness	Kurtosis	Outliers	*p*	Skewness	Kurtosis
**Normally** **Distributed Data Thresholds**	**≥0.05**	**−1 < γ < 1**	**−1 < κ < 1**	**n = 0**	**≥0.05**	**−1 < γ < 1**	**−1 < κ < 1**
**Average**	<0.001	−0.677	5.664	n = 12	0.303	0.157	0.035
**Local Maxima**	<0.001	−0.807	4.605	n = 7	0.643	−0.084	−0.012
**Local Minima**	<0.001	0.473	1.300	n = 8	0.363	−0.099	−0.162

**Table 2:** Linear regression metrics across activities.

Activity	R^2^	Slope	Intercept
**Ideal**	**1.0**	**1.00**	**±0.0°**
**Sit to Stand**	0.96	1.10	−8.1°
**Standing Knee Bend**	0.97	1.04	−0.5°
**Treadmill Gait**	0.86	0.98	+1.8°
**Heel Slides**	0.95	1.04	−3.7°
**Seated March**	0.93	1.02	−2.4°
**Long Arc Quad**	0.97	1.09	−7.3°
**Wall Slide**	0.92	1.02	−1.4°
**TUG Test**	0.93	1.06	−4.6°

## Data Availability

Due to the volume of data collected, data are available on a case-by-case basis. Please contact the corresponding author for data access requests.

## Abbreviations

The following abbreviations are used in this manuscript:

ROM	Range of motion
TKA	Total knee arthroplasty
MOCAP	Motion capture
NJ	New Jersey
IMU	Inertial measurement unit
ANOVA	Analysis of variance
OA	Osteoarthritis
ADL	Activity of daily living
IRB	Institutional review board
BMI	Body mass index
FL	Florida
CR	Cruciate retaining
QTM	Qualisys track manager
V3D	Visual3D
DOF	Degree of freedom
3D	Three dimensions
AIM	Automatic identification of markers
TUG	Timed up and go
S2S	Sit to stand
SKB	Standing knee bends
HS	Heel slides
SM	Seated march
LAQ	Long arc quadriceps exercise
WS	Wall slides
